# The efficacy of duloxetine, non-steroidal anti-inflammatory drugs, and opioids in osteoarthritis: a systematic literature review and meta-analysis

**DOI:** 10.1186/1471-2474-15-76

**Published:** 2014-03-11

**Authors:** Julie Myers, Ronald C Wielage, Baoguang Han, Karen Price, James Gahn, Marie-Ange Paget, Michael Happich

**Affiliations:** 1 Medical Decision Modeling, Inc, 8909 Purdue Road, Suite 550, Indianapolis, IN, USA; 2 Eli Lilly and Company, Indianapolis, IN, USA; 3 Lilly France, Neuilly sur Seine, France; 4 Lilly Deutschland GmbH, Bad Homburg, Germany

**Keywords:** Duloxetine, Osteoarthritis, Meta-analysis, NSAID, Opioid, WOMAC

## Abstract

**Background:**

This meta-analysis assessed the efficacy of duloxetine versus other oral treatments used after failure of acetaminophen for management of patients with osteoarthritis.

**Methods:**

A systematic literature review of English language articles was performed in PUBMED, EMBASE, MedLine In-Process, Cochrane Library, and ClinicalTrials.gov between January 1985 and March 2013. Randomized controlled trials of duloxetine and all oral non-steroidal anti-inflammatory drugs and opioids were included if treatment was ≥12 weeks and the Western Ontario and McMaster Universities Index (WOMAC) total score was available. Studies were assessed for quality using the assessment tool from the National Institute for Health and Clinical Excellence guidelines for single technology appraisal submissions.

WOMAC baseline and change from baseline total scores were extracted and standardized. A frequentist meta-analysis, meta-regression, and indirect comparison were performed using the DerSimonian-Laird and Bucher methods. Bayesian analyses with and without adjustment for study-level covariates were performed using noninformative priors.

**Results:**

Thirty-two publications reported 34 trials (2 publications each reported 2 trials) that met inclusion criteria. The analyses found all treatments except oxycodone (frequentist) and hydromorphone (frequentist and Bayesian) to be more effective than placebo. Indirect comparisons to duloxetine found no significant differences for most of the compounds. Some analyses showed evidence of a difference with duloxetine for etoricoxib (better), tramadol and oxycodone (worse), but without consistent results between analyses. Forest plots revealed positive trends in overall efficacy improvement with baseline scores. Adjusting for baseline, the probability duloxetine is superior to other treatments ranges between 15% to 100%.

Limitations of this study include the low number of studies included in the analyses, the inclusion of only English language publications, and possible ecological fallacy associated with patient level characteristics.

**Conclusions:**

This analysis suggests no difference between duloxetine and other post-first line oral treatments for osteoarthritis (OA) in total WOMAC score after approximately 12 weeks of treatment. Significant results for 3 compounds (1 better and 2 worse) were not consistent across performed analyses.

## Background

Over 50 treatment modalities for osteoarthritis (OA) of the hip and knee have been evaluated by the Osteoarthritis Research Society International (OARSI) [1,2]. Oral pharmacologic modalities included acetaminophen, non-steroidal anti-inflammatory drugs (NSAIDs), and both strong and weak opioids. Guidelines have recommended acetaminophen for first-line use, with NSAIDs and opioids as second and third lines of treatment [1,3-5]. However, reservations have been expressed concerning the long-term safety and efficacy of NSAIDs and opioids [1,2,5,6]. Some reviews have gone further and recommended against their long-term use [7,8]. Recently published meta-analyses suggest that currently available oral treatments have only limited efficacy in the average patient with OA [6]. In addition, the efficacy seen in trials seems to be impacted by trial design and baseline factors and may be limited to the first few weeks of use [6].

Earlier meta-analyses have primarily focused on pain and have not assessed broader functioning. They have predominantly investigated single-substance classes, included both short- and long-term trials, and sometimes encompassed both OA and other chronic pain indications [7-25]. Also, these analyses could not include evidence for substances that were unavailable when they were performed, such as duloxetine, a newly available treatment option in the US.

Duloxetine is a selective serotonin and norepinephrine reuptake inhibitor (SNRI) that has demonstrated efficacy in OA in Phase III clinical trials as well as a favorable adverse event profile across indications [26-28]. Duloxetine is thought to inhibit pain through its enhancement of serotonergic and noradrenergic activity in the central nervous system. It is currently indicated in the US for the management of pain disorders, including diabetic peripheral neuropathic pain (DPNP), fibromyalgia, and chronic musculoskeletal pain due to OA and chronic low back pain [29].

We conducted a systematic literature review followed by a meta-analysis to assess the efficacy of duloxetine versus other commonly used post first-line OA treatments, including NSAIDs and opioids. Our study reflected the chronic nature of OA by including only trials of 12 or more weeks duration (the recommended duration for confirmatory trials) [30] and a more inclusive set of OA symptoms by using the Western Ontario MacMaster Universities Osteoarthritis Index (WOMAC), which includes subscales for function and stiffness as well as pain [31]. We also sought to confirm the influence of design and baseline factors observed in a recent OA meta-analysis [6]. Both frequentist and Bayesian analyses were undertaken to assess the effect of duloxetine compared to the other available oral treatments.

## Methods

### Inclusion and exclusion criteria

Randomized controlled trials (RCTs) were included for OA treatment with duloxetine, NSAIDs or opioids at dosages consistent with United Kingdom prescribing information [32]. All included studies were of at least 12 weeks duration and published in English. Articles were included if they evaluated clinical efficacy using WOMAC total scores. Studies were excluded that did not report clinical efficacy of OA, and did not have at least 2 arms of a treatment of interest, or 1 arm of a treatment of interest and a placebo arm.

When it was unclear from the title or abstract whether a study met the criteria, the full paper was acquired and read. Determination of inclusion/exclusion was performed by 2 persons working independently. When their conclusions were not in agreement the persons met and came to a consensus.

### Literature search

The literature search was performed on all articles published between January 1985 and March 2013 in PUBMED, EMBASE, MEDLINE In-Process & Other Non-Indexed Citations, Cochrane Central Register of Controlled Trials, Cochrane Database of Systematic Reviews, and ClinicalTrials.gov. The search conducted in PUBMED used the following terms: (ibuprofen OR naproxen OR diclofenac OR meloxicam OR etoricoxib OR celecoxib OR mefenamic OR indometacin OR etodolac OR tramadol OR morphine OR codeine OR dihydrocodeine OR oxycodone OR diamorphine OR methadone OR hydromorphone OR duloxetine) AND (osteoarthritis) AND (English [lang]) AND (clinical trial [ptyp]). The search conducted in the other databases used the same search terms, but without the specific limitation of clinical trial publication type.

### Data extraction

Data extraction was performed by 1 reviewer and checked by a second reviewer using a predefined data extraction form. Discrepancies were resolved by discussion between reviewers. For each study, reviewers extracted data that were deemed to potentially impact efficacy outcomes, such as study population (percent women, mean age, mean duration of OA), study design (duration, washout period, flare requirement, concomitant analgesic use, enriched enrollment, missing imputation technique), and outcomes (WOMAC score at baseline, endpoint, and change from baseline with measures of variance). Studies were categorized as having a washout period if the publication mentioned a period of washout or no treatment before randomization. A study was classified as requiring flare if the publication stated that after the washout/no treatment period patients were required to exhibit a flare of symptoms to continue in the study. Studies were classified as allowing concomitant analgesic use if patients could use analgesic medications in addition to their assigned treatment throughout the study; rescue medication was not considered concomitant use.

For studies that did not report sufficient data to be included in the analysis, 3 attempts were made to contact authors by email to obtain missing information. Studies were assessed for quality using the assessment tool from the National Institute for Health and Clinical Excellence (NICE) guidelines for Single Technology Appraisal submissions [33]. This 7-item questionnaire evaluates each trial based on randomization, adequate concealment of treatment allocation, similarities between treatment groups, degree of blinding, balance of withdrawals and dropouts between treatment groups, reporting of all outcomes measured, and use of intention to treat analyses. Studies were assessed by one reviewer and independently checked by a second reviewer. Positive responses were tallied for a total possible score of 7, with higher scores representing better quality.

### Outcome measure

The outcome measure for the meta-analysis was the change from baseline total WOMAC score as reported at 12 or more weeks. The WOMAC instrument consists of 24 questions answered on a 0–4 Likert or 0–100 visual analogue scale (VAS). The WOMAC has 3 subscales: function (17 questions), pain (5 questions), and stiffness (2 questions). A lower WOMAC score indicates fewer symptoms, thus improvement is shown as a negative value; negative values of larger magnitude are indicative of greater efficacy. WOMAC total and subscale scores are reported inconsistently, with publications reporting scores on different scales, some subscale scores and not others, different measures of variance, or no measures of variance. Scores are commonly reported as: a) a total of the Likert scores, b) a total of the VAS scores, or c) normalized units with total and subscale scores reported on 0–100 scales [34]. To overcome this issue, WOMAC total scores were converted to a 0-100 normalized scale using a direct ratio. If change from baseline was not reported, it was calculated as the difference between baseline and endpoint or, if not possible, as the difference between baseline and a weighted average of multiple observations during treatment [35]. When subscale scores were reported without the total score, the total score and variance were calculated from the subscales. Missing stiffness subscale scores were imputed by substituting the mean of those reported for that treatment. Studies reporting neither the total score nor the pain and function subscale scores were omitted from the analysis.

### Statistical analysis

Frequentist and Bayesian methods were used to assess the effect of including the direct and indirect data in the analysis. The frequentist meta-analysis using Bucher indirect comparisons was chosen because it reports traditional statistical measures, whereas the Bayesian network meta-analysis allows for inclusion of both direct and indirect information in a single step. In both frequentist and Bayesian methods, if multiple arms for a treatment were present in a study at different doses, the arms used were consistent with the United Kingdom prescribing information. For tramadol, the 400-mg daily dose was not included as it is associated with higher rates of adverse events and similar efficacy to the 300-mg dose [36].

The frequentist meta-analysis used the difference between treatment and placebo of the change from baseline WOMAC score for each active treatment. Random effects models using the DerSimonion-Laird method were employed regardless of heterogeneity due to study design and population dissimilarities [37]. Estimated treatment effects compared to placebo and compared to duloxetine were calculated with their 95% confidence intervals using the Bucher method of indirect comparison [38-41]. Frequentist analyses were performed with Comprehensive Meta-Analysis software (CMA; Biostat, Englewood NJ) [42]. Publication bias was assessed by funnel plot with Duval and Tweedie’s trim and fill [37].

Random effects Bayesian network meta-analyses were performed using the change from baseline score for all available studies. Bayesian methods described in NICE Decision Support Unit documents were modified to accommodate continuous data analysis [43,44]. Each trial’s specific relative treatment effect was assumed to be drawn from a random effects normal distribution with a common random effects variance for all treatment comparisons. The best model was selected based on the deviance information criteria (DIC), described in Cooper et al. [45] and Dias et al. [46], and standard deviation (SD), which provide measures of model fit. The consistency between direct and indirect evidence was performed using node splitting methods described by Dias et al. [46]. Estimated treatment effects compared to placebo and duloxetine were given with their associated 95% credible intervals as well as the probability of the treatment being superior to duloxetine. Sensitivity analyses were run on various scenarios, including adjustment for baseline scores, flare requirement, and analgesic use. The Bayesian analyses were conducted using WinBUGS version 1.4.3 (MRC Biostatistics Unit; Cambridge, UK) [47].

Heterogeneity was assessed by calculating the I^2^ statistic. Twelve population and study characteristics were assessed as possible confounding factors by visually inspecting forest plots for the magnitude and variability of study WOMAC scores. These characteristics included washout period [yes/no], enriched enrollment [yes/no], flare required [yes/no], chronic pain definition [<6 months/> = 6 months], baseline pain level, concomitant analgesic use allowed [yes/no], missing imputation technique, quality assessment, study mean age, study mean duration of OA, site of OA, and the percent women. When forest plots suggested a possible relationship, both frequentist and Bayesian meta-regression were conducted to account for heterogeneity of treatment effect. Bayesian methods assumed the same covariate effect for all active treatments. Noninformative priors were used for all parameters; a uniform distribution was used for random effects variance and normal distributions with very large variance for all other parameters, including treatment effect and covariate effect.

## Results

### Literature search

**Figure 1:**
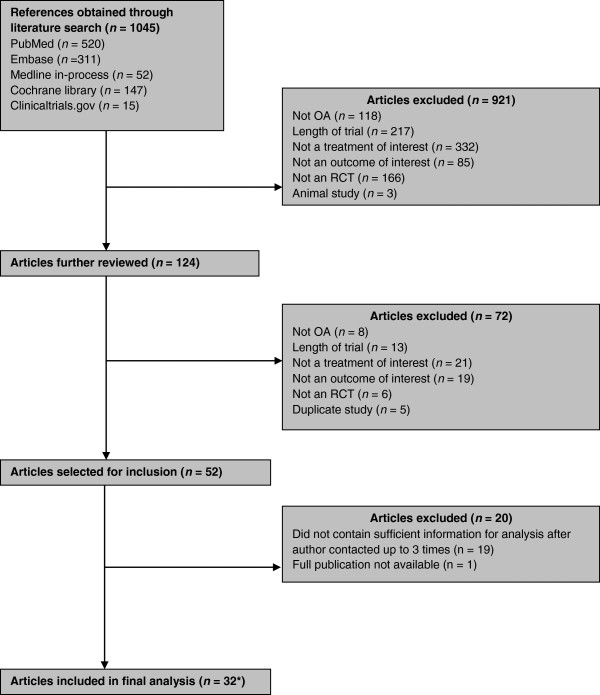
**Article selection flow chart.** *Reporting 34 studies.

Figure 1 provides a flow diagram of the article selection process. Of the initial 1045 articles identified, 124 met the eligibility criteria for possible inclusion based on abstract review. Most excluded studies lacked a treatment of interest or the duration was too short. Thirty-two articles with 47 active treatment arms reported sufficient information to be included in the meta-analysis, for a total number of 17,442 patients (mean age 60.3 years, 64.9% women). Sixteen articles were found for celecoxib, 9 for naproxen, 5 each for tramadol and etoricoxib, 3 for duloxetine, and 2 each for ibuprofen, hydromorphone and oxycodone. Of the 20 other studies identified in the literature search, the most frequent reason for exclusion was incomplete reporting of WOMAC scores, especially the omission of a measure of variance. One full paper was unavailable [48].

**Figure 2:**
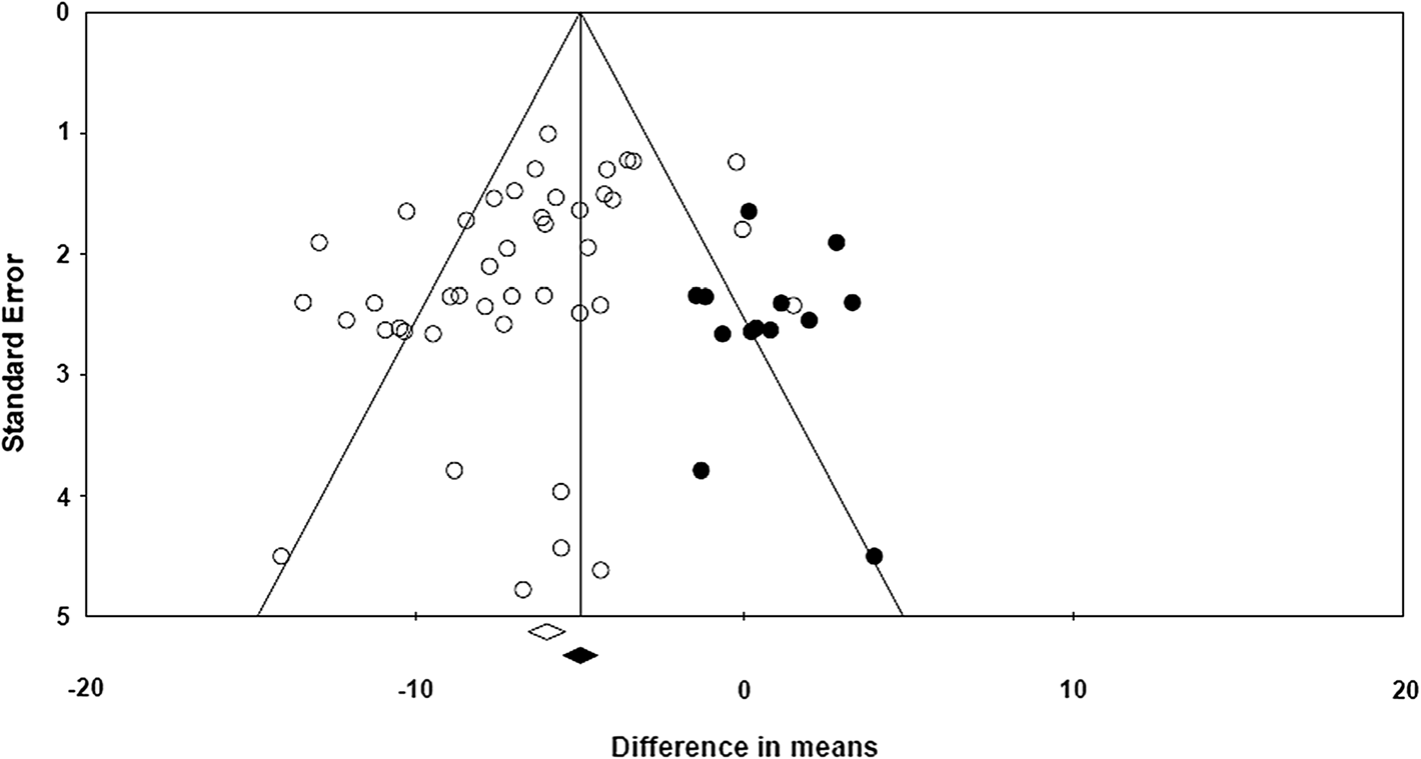
**Funnel plot of standard error by difference in mean.** Note: o = actual publication; ● = hypothetical omitted study.

Table 1 presents the studies included in the meta-analysis with 5 extracted study characteristics as well as baseline and change from baseline WOMAC scores. The duration of nearly all studies was 12 to13 weeks, with a range of 12 to 26 weeks. The size of treatment arms ranged from 51 patients in a placebo arm to 481 in a celecoxib arm. Seven studies did not report baseline WOMAC scores. Three studies were identified in which complete WOMAC scores were not reported in the publication, but were available on clinicaltrials.gov. These studies are identified in the table with both the publication reference and the NTC number from clinicaltrials.gov. Table 2 presents descriptive statistics of the included studies grouped by treatment. In Table 3 the quality assessments of the included studies are presented. Of the 32 included articles, 26 (81%) had a quality score of 6 or 7 (maximum score 7) and the other 6 studies had a quality score of 5, indicating that the included studies were of sufficiently high quality. A funnel plot assessing publication bias, run on all studies as not enough studies per compound were available, was roughly symmetrical, with slightly more studies on the left, indicating little effect of publication bias on the results of this analysis (Figure 2). Missing publications have been imputed using Duval and Tweedie’s trim and fill and appear as solid points among the actual publications depicted as circles [37]. This method suggests that possible missing studies would trend to non-significant differences in means.

**Table 1:** **Characteristics of all included studies** (**Alphabetically ordered**)

**Study**	**Treatment**	**n**	**Mean age (yrs)**	**Baseline WOMAC score (SD)**	**Change from baseline WOMAC score (SD)**	**Percentage women**	**Duration OA at baseline (yrs)**	**Flare required**	**Concomitant analgesic allowed**^**d**^	**OA site**
Abou-Raya et al. 2012 [49]^b^	Duloxetine	144	68.9	50.63 (9.56)	-12.40 (14.02)	16	5.7	No	Yes	Knee
	Placebo	144	68.5	50.94 (9.47)	-3.96 (15.24)	17	5.6			
Afilalo et al. 2010 [50]^f^	Oxycodone 40–100 mg	342	58.2		-27.50 (21.75)	59		No	Not mentioned	Knee
Study NCT00421928	Placebo	337	58.2		-22.50 (21.00)	59				
Baerwald et al. 2010 [51]^b^	Naproxen 1000 mg	156	62.26		-22.54 (20.40)	32.1		Yes	No	Hip
	Placebo	331	63.29		-14.80 (22.27)	37.2				
Bensen et al. 1999 [52]	Celecoxib 200 mg	202	63	53.13 (17.08)	-12.50 (18.06)	72	9	No	No	Knee
	Naproxen 1000 mg	198	62	55.10 (14.58)	-12.40 (18.91)	71	10			
	Placebo	203	62	53.85 (15.42)	-6.35 (16.18)	75	11			
Bingham et al. 2007a [53]	Etoricoxib 30 mg	231	62.1	65.40 (13.03)	-24.37 (21.37)	66.2		Yes	No	Hip/knee
	Celecoxib 200 mg	241	62.5	66.20 (13.24)	-22.21 (21.66)^a^	69.7				
	Placebo	127	62.8	64.67 (13.30)	-10.98 (22.14)^a^	65.4				
Bingham et al. 2007b [53]	Etoricoxib 30 mg	244	61.9	67.23 (13.24)	-24.37 (22.19)	69.7		Yes	No	Hip/knee
	Celecoxib 200 mg	247	62.2	65.59 (14.59)	-23.19 (23.29)^a^	61.9				
	Placebo	117	60.9	64.98 (13.81)	-12.29 (22.63)^a^	65				
Boswell et al. 2008 [54]	Celecoxib 200 mg	185	59.7	63.55 (14.70)	-23.46 (24.51)^a^	67	8.8	No	No	Knee
	Placebo	186	60.5	63.37 (13.68)	-18.46 (23.46)^a^	73	8			
Burch et al. 2007 [55]^f^	Tramadol 200–300 mg	432	62		34.96 (14.74)^g^	64		Yes	No	Knee
Study NCT00833794	Placebo	214	62		35.20 (15.13)^g^	62				
Chappell et al. 2011 [26]^b^	Duloxetine 60–120 mg	128	63.16	51.63 (10.45)	-20.50 (11.87)	70	8.14	No	Yes	Knee
Study NCT00433290	Placebo	128	61.9	53.82 (9.04)	-16.25 (12.26)	84	6.74			
Chappell et al. 2009 [27]^b^	Duloxetine 60–120 mg	111	62.07	57.10 (12.15)	-24.01 (16.07)	63.06	9.04	No	Yes	Knee
Study NCT00408421	Placebo	120	62.48	56.51 (11.12)	-16.81 (13.67)	67.5	9.3			
Clegg et al. 2006 [56]^b, e^	Celecoxib 200 mg	318	59.4	47.10 (13.36)	-17.95 (14.98)	66.7	10.1	No	Yes	Knee
	Placebo	313	58.2	46.23 (13.49)	-14.58 (15.99)	63.9	9.5			
DeLemos et al. 2011 [57]	Tramadol 200 mg	199	62	61.13 (14.02)	-16.24 (24.22)	62.3	8.5	No	No	Hip/knee
	Tramadol 300 mg	199	59.7	60.37 (15.93)	-22.10 (24.16)	61.8	7.6			
	Celecoxib 200 mg	202	60	58.21 (15.26)	-25.60 (24.58)	64.9	8			
	Placebo	200	58.9	59.95 (15.49)	-17.73 (24.28)	68.5	7.8			
Essex et al. 2012 [58]^c^	Celecoxib 200 mg	296	60	56.15 (15.42)	-23.13 (19.71)	64.9	7.2	Yes	No	Knee
	Naproxen 1000 mg	293	60.7	56.56 (15.73)	-23.54 (19.61)	67.6	8.5			
Fishman et al. 2007 [59]	Tramadol 200 mg	107	61	58.42 (13.99)	-24.39 (21.19)^a^	59.8		No	Yes	Knee
	Tramadol 300 mg	105	60	63.74 (15.21)	-25.54 (44.61)	65.7				
	Placebo	224	61	61.29 (14.16)	-18.82 (38.32)	61.6				
Fleischmann et al. 2006 [60]^b^	Celecoxib 200 mg	444	61.3	50.94 (16.76)	-16.67 (18.95)	67.1	6.7	No	No	Knee
	Placebo	231	61.5	48.65 (16.97)	-9.69 (16.82)	66.2	6.6			
Fleischmann et al. 2001 [61]^b^	Tramadol 200–400 mg	63	62.52		41.60 (20.50)^g^	65.1	7.94	Yes	No	Knee
	Placebo	66	62.45		50.40 (22.50)^g^	59.1	7.76			
Gana et al. 2006 [36]	Tramadol 200 mg	201	59.1	63.77 (13.14)	-21.25 (23.92)	63.7	7.7	No	No	Hip/knee
	Tramadol 300 mg	201	58.5	60.10 (14.73)	-20.27 (23.81)	59.2	8			
	Placebo	205	56.4	61.82 (14.82)	-14.19 (23.45)	68.8	7.7			
Hochberg et al. 2011a [62]	Celcoxib 200 mg	242	33.2		-5.56 (40.51)^a, h^	61.2		Yes	Yes	Knee
	Placebo	124	32.7			66.1				
Hochberg et al. 2011b [62]	Celecoxib 200 mg	244	33		-4.36 (41.89)^a, h^	62.7				Knee
	Placebo	122	33			63.1				
Kivitz et al. 2002 [63]	Naproxen 1000 mg	205	60.4	55.91	-18.79 (19.78)	63	9.4	No	No	Knee
	Placebo	205	60.3	55.72	-14.04 (19.71)	64	8.3			
Kivitz et al. 2001 [64]	Celecoxib 200 mg	207	62	52.29 (16.73)	-10.10 (15.92)	65	7.2	Yes	No	Hip
	Naproxen 1000 mg	207	64	51.88 (17.24)	-11.98 (16.07)	66	7.3			
	Placebo	218	64	52.81 (15.60)	-4.38 (15.70)	67	7.9			
Lehmann et al. 2005 [65]^b^	Celecoxib 200 mg	420	62.9	52.60 (14.93)	-15.31 (16.47)	68.3	4.4	Yes	yes	Knee
	Placebo	424	61.7	51.77 (15.09)	-11.77 (19.03)	71.9	3.9			
Leung et al. 2002 [66]	Etoricoxib 60 mg	224	62.93	63.84 (13.89)	-22.19 (15.91)	77.2	5.88	Yes	No	Hip/knee
	Naproxen 1000 mg	221	63.16	63.76 (13.36)	-21.91 (15.81)	78.3	6.25			
	Placebo	56	64.09	68.11 (10.83)	-13.26 (15.17)	82.1	6.3			
Markenson et al. 2005 [67]^b, e^	Oxycodone 10–120 mg	56	62	64.70 (15.71)^a^	-14.93 (26.09)	68		No	Yes	Hip/knee/spine/other
	Placebo	51	64	63.80 (15.00)	-0.87 (19.72)	78				
Puopolo et al. 2007 [68]	Etoricoxib 30 mg	224	62.1	64.95	-24.90 (23.14)	77.7	6.6	Yes	Yes	Hip/knee
	Ibuprofen 2400 mg	213	62.3	63.18	-21.73 (22.49)	73.7	6.7			
	Placebo	111	64	64.56	-14.43 (21.23)	75.7	6.5			
Rauck et al. 2013 [69]	Hydromorphone 16 mg	330	59.5		-17.00 (19.98)	64.2		No	Yes	Hip/knee
	Placebo	331	60		-13.00 (20.01)	63				
Schnitzer et al. 2011 [70]^b^	Celecoxib 200 mg	419	61.7	54.90 (14.49)	-16.58 (15.24)^a^	61.3	3.7	No	No	Hip
	Placebo	416	61.4	54.58 (15.11)	-10.62 (13.83)^a^	60.6	3.8			
Schnitzer et al. 2011 [71]^b^	Naproxen 1000 mg	254	60		-26.29 (18.71)^a^	70.5		Yes	No	Knee
	Placebo	257	60.15		-16.04 (18.62)^a^	72.65				
Schnitzer et al. 2010 [72]^b^	Naproxen 1000 mg	227	61.1	70.08 (12.98)	-33.33 (20.23)^a^	67.4		Yes	No	Knee
	Placebo	221	61	69.85 (13.12)	-20.42 (20.17)^a^	71.9				
Sheldon et al. 2005 [73]^b^	Celecoxib 200 mg	393	60.2	54.79 (15.45)	-16.25 (19.08)	63.1	6.7	No	No	Knee
	Placebo	382	60.8	55.31 (14.36)	-9.90 (17.01)	61.3	7			
Sowers et al. 2005 [74]^c^	Celecoxib 200 mg	136	61.8	46.20 (22.16)	-16.30 (20.99)	62		No	No	Hip/knee
	Naproxen 1000 mg	128	63.6	51.40 (20.36)	-14.70 (21.50)	60				
Tannenbaum et al. 2004 [75]^b^	Celecoxib 200 mg	481	64.1	50.73 (16.04)	-13.96 (16.46)	69.2	5.3	No	No	Knee
	Placebo	243	64.6	51.25 (14.58)	-9.79 (16.77)	67.1	4.3			
Vojtassak et al. 2011 [76]	Hydromorphone	138	65	60.00 (10.11)	-17.75 (14.62)	77		No	Yes	Hip/knee
	Placebo	149	66	57.92 (10.36)	-17.69 (15.79)	68				
Wiesenhutter et al. 2005 [77]	Etoricoxib 30 mg	214	63.1	68.68 (16.64)	-24.52 (22.97)	70.1	7.9	Yes	No	Hip/Knee
	Ibuprofen 2400 mg	210	61.3	68.13 (17.02)	-23.65 (23.13)	70	8.2			
	Placebo	104	59.5	69.71 (16.52)	-14.20 (20.24)	72.1	6.9			

Note: ^a^value imputed by estimating a stiffness subscore from other scores reported for that treatment; ^b^study longer than 12 weeks duration; ^c^included in Bayesian analysis only, no placebo arm, ^d^washout is not considered as complete in studies with concomitant analgesic use; ^e^denotes studies without a washout period; ^f^denotes studies with enriched enrollment design; ^g^indicates endpoint WOMAC score, change from baseline not available in these studies; ^h^indicates difference from placebo in WOMAC score change from baseline.

**Table 2:** Study descriptive statistics by treatment

**Treatment**	**Total n**	**Mean age (yrs)**	**Mean percentage women**	**Mean duration of OA (yrs)**
Duloxetine	383	65.00	73.25	7.48
Ibuprofen	423	61.80	71.86	7.44
Naproxen	1889	61.73	68.41	8.26^a^
Celecoxib	4681	61.63	65.60	6.58^a^
Etoricoxib	1137	62.41	72.12	6.78^a^
Tramadol	1507	60.60	62.67	7.95^a^
Oxycodone	398	58.73	60.27	NR
OROS hydromorphone	468	59.5^b^	67.97	NR
Placebo	6560	61.26^b^	66.97	6.78^a^

^a^not all studies reported duration of OA; ^b^one study did not report mean age; *NR* = not reported.

**Table 3:** Quality assessment of included articles

**Study**	**Randomization appropriate**^**a**^	**Treatment allocation concealment**^**b**^	**Groups similar at baseline**^**c**^	**Blinding of all participants**^**d**^	**Unexpected imbalance in drop**-**outs**^**e**^	**Measured outcomes not reported**^**f**^	**ITT analysis, missing data**^**g**^	**Quality score**^**h**^
Abou-Raya et al. 2012 [49]	Yes	Yes	Yes	Yes	No	No	Yes	7
Afilalo et al. 2010 [50]	Yes	Yes	Yes	Yes	No	No	Yes	7
Baerwald et al. 2010 [51]	Not clear	Not clear	Yes	Yes	No	No	Yes	5
Bensen et al. 1999 [52]	Yes	Yes	Yes	Yes	No	No	Yes	7
Bingham et al. 2007 [53]	Not clear	Not clear	Yes	Yes	No	No	Yes	5
Boswell et al. 2008 [54]	Not clear	Not clear	Yes	Yes	No	No	Yes	5
Burch et al. 2007 [55]	Yes	Yes	Yes	Yes	No	No	Not clear	6
Chappell et al. 2011 [26]	Yes	Yes	Yes	Yes	No	No	Yes	7
Chappell et al. 2009 [27]	Yes	Yes	Yes	Yes	No	No	Yes	7
Clegg et al. 2006 [56]	Yes	Yes	Yes	Yes	No	No	Yes	7
DeLemos et al. 2011 [57]	Not clear	Yes	Yes	Yes	No	No	Yes	6
Essex et al. 2012 [58]	Yes	Yes	Yes	Yes	No	No	Yes	7
Fishman et al. 2007 [59]	Yes	Yes	Yes	Yes	Yes, explained	No	Yes	6
Fleischmann et al. 2006 [60]	Not clear	Yes	Yes	Yes	No	No	Yes	6
Fleischmann et al. 2001 [61]	Yes	Yes	Yes	Yes	No	No	Yes	7
Gana et al. 2006 [36]	Yes	Yes	Yes	Yes	No	No	Yes	7
Hochberg et al. 2011 [62]	Yes	Yes	Yes	Yes	No	No	Yes	7
Kivitz et al. 2002 [63]	Yes	Yes	Yes	Yes	No	No	Yes	7
Kivitz et al. 2001 [64]	Yes	Yes	Yes	Yes	No	No	Yes	7
Lehmann et al. 2005 [65]	Yes	Yes	Yes	Yes	No	No	Yes	7
Leung et al. 2002 [66]	Yes	Yes	Yes	Yes	No	Yes	Not clear	5
Markenson et al. 2005 [67]	Yes	Yes	Yes	Yes	No	No	Yes	7
Puopolo et al. 2007 [68]	Yes	Not clear	Yes	Yes	No	No	Not clear	5
Rauck et al. 2013 [69]	not clear	Yes	Yes	Yes	No	No	Yes	6
Schnitzer et al. 2011 [70]	Yes	Yes	Yes	Yes	No	No	Yes	7
Schnitzer et al. 2011 [71]	Not clear	Not clear	Yes	Yes	No	No	Yes	5
Schnitzer et al. 2010 [72]	Yes	Not clear	Yes	Yes	No	No	Yes	6
Sheldon et al. 2005 [73]	Yes	Yes	Yes	Yes	No	No	Yes	7
Sowers et al. 2005 [74]	Yes	Not clear	Yes	Yes	No	No	Yes	6
Tannenbaum et al. 2004 [75]	Not clear	Yes	Yes	Yes	No	No	Yes	6
Vojtassak et al. 2011 [76]	Yes	Yes	Yes	Yes	No	No	Yes	7
Wiesenhutter et al. 2005 [77]	Not clear	Yes	Yes	Yes	No	No	Yes	6

^a^“Was randomisation carried out appropriately?”.

^b^“Was the concealment of treatment allocation adequate?”.

^c^“Were the groups similar at the outset of the study in terms of prognostic factors, for example, severity of disease?”.

^d^“Were the care providers, participants and outcome assessors blind to treatment allocation? If any of these people were not blinded, what might be the likely impact on the risk of bias (for each outcome)?”.

^e^“Were there any unexpected imbalances in drop-outs between groups? If so, were they explained or adjusted for?”.

^f^“Is there any evidence to suggest that the authors measured more outcomes than they reported?”.

^g^“Did the analysis include an intention-to-treat analysis? If so, was this appropriate and were appropriate methods used to account for missing data?”.

^h^Quality Score is calculated by summing the positive answers to each question (“yes” answers to questions 1–4 and 7, and “no” answers to questions 5 &6).

### Statistical results

Results of both the frequentist and Bayesian analyses are shown in Table 4. The frequentist approach analyzed 32 of the 34 studies, excluding Sowers et al. [74] and Essex et al. [58] due to the lack of placebo arms. All active treatments, except hydromorphone and oxycodone, were found to statistically improve the WOMAC total score compared to placebo. Indirect comparisons to duloxetine using the Bucher method found all confidence intervals but etoricoxib encompassed zero, indicating the differences between duloxetine and all treatments except etoricoxib were not statistically significant. Two compounds, ibuprofen and etoricoxib, had an I^2^ of zero while naproxen, celecoxib, duloxetine, oxycodone, hydromorphone, and tramadol had I^2^s of 52%, 33%, 44%,72%, 64%, and 58%, respectively, indicating substantial heterogeneity [78,79]. However, the direction of the treatment effect was the same for all but one study; the magnitude of the treatment effect in these studies was the source of heterogeneity.

**Table 4:** **Indirect comparison**: **results for WOMAC total score change from baseline**

	**Duloxetine**	**Ibuprofen**	**Naproxen**	**Celecoxib**	**Etoricoxib**	**Tramadol**	**Oxycodone**	**Hydromorphone**
**Frequentist analysis**								
Number of studies	3	2	7^f^	14^f^	5	5	2	2
Change from baseline vs. placebo, mean	-6.48	-8.34	-8.27	-5.78	-11.04	-3.99	-8.56	-2.13
95% CI	[-9.09, -3.88]	[-11.98, -4.71]	[-10.27, -6.28]	[-6.86, -4.69]	[-13.24, -8.84]	[-6.74, -1.23]	[-17.23, 0.11]	[-5.99, 1.72]
I^2^ (%)	44.35	0	51.92	32.49	0	58.03	71.99	63.54
Indirect vs. Duloxetine ^a^	NA	-1.86	-1.93	0.71	-4.56	2.36	-2.07	4.35
95% CI^b^	NA	[-6.33, 2.62]	[-4.70, 0.84]	[-2.12, 3.53]	[-7.97, -1.15]	[-1.00, 5.73]	[-11.13, 6.98]	[-0.31, 9.01]
**Bayesian analysis**								
Number of studies contributing to each compound^c^	3	2	9	16	5	5	2	2
Change from baseline vs. placebo, mean^d^	-6.47	-7.85	-7.9	-6.2	-9.53	-2.89	-7.04	-2.19
95% CI	[-9.27, -3.7]	[-11.59, -4.18]	[-9.54, -6.27]	[-7.46, -5.03]	[-11.86, -7.3]	[-5.41, -0.54]	[-11.35, -2.95]	[-5.52, 1.21]
Indirect vs. Duloxetine^a^	NA	-1.38	-1.43	0.27	-3.07	3.57	-0.58	4.28
95% CI^b^	NA	[-6.04, 3.21]	[-4.65, 1.81]	[-2.78, 3.28]	[-6.66, 0.49]	[-0.17, 7.19]	[-5.69, 4.32]	[-0.01, 8.69]
*Probability Duloxetine is Superior*	*NA*	*0.28*	*0.19*	*0.57*	*0.04*	*0.97*	*0.41*	*0.97*
Number of studies contributing to each compound for adjusted for baseline WOMAC score^e^	3	2	7	14	5	3	1	1
Indirect vs. Duloxetine adjusted for baseline WOMAC score^e^	NA	1.85	0.24	0.83	-0.43	4.92	-4.67	8.19
95% CI^b^	NA	[-2.13, 5.9]	[-2.36, 2.87]	[-1.45, 3.14]	[-3.4, 2.57]	[1.51, 8.34]	[-13.24, 4.07]	[3.84, 12.56]
*Probability Duloxetine is Superior*	*NA*	*0.82*	*0.57*	*0.76*	*0.38*	*1*	*0.15*	*1*

^a^A positive (negative) result indicates that the compared treatment is worse (better) than duloxetine.

^b^If zero does not fall between the upper and lower bounds the null hypothesis (treatments are the same) is rejected.

^c^There are fewer studies in the adjusted analyses.

^d^Random effects model.

^e^Random effects model adjusting for baseline excluding trials with no baseline.

^f^2 studies without placebo arms were not included in the frequentist analysis.

**Figure 3:**
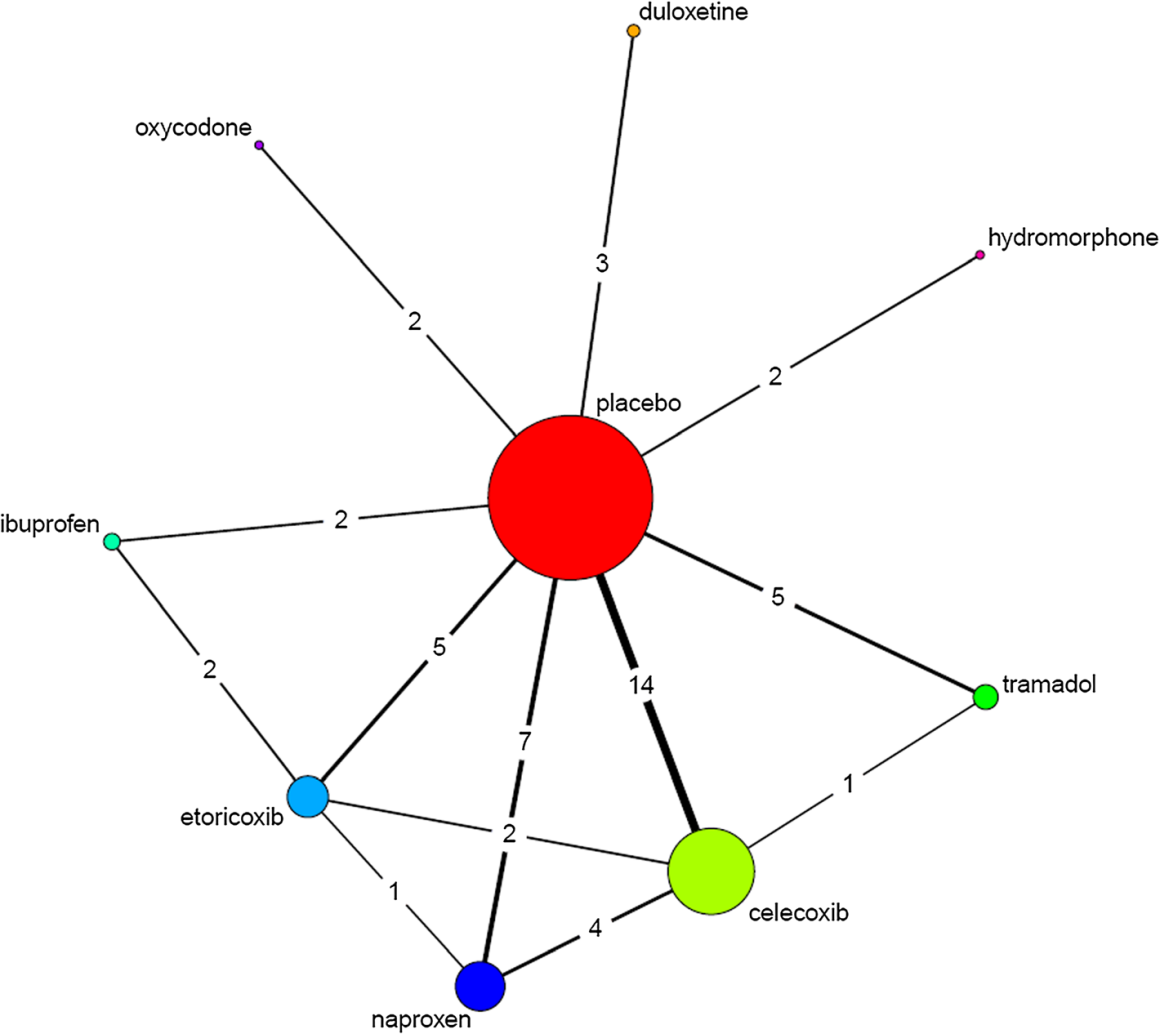
**Network of evidence including direct and indirect comparisons.** Note: the numbers represent number of comparisons between treatments.

The Bayesian network meta-analysis included all 34 studies. Figure 3 depicts the network of direct and indirect evidence. As shown in Table 4, the results lead to similar conclusions as the frequentist results, as all 95% credible intervals of the difference between duloxetine and active treatments included zero.

**Figure 4:**
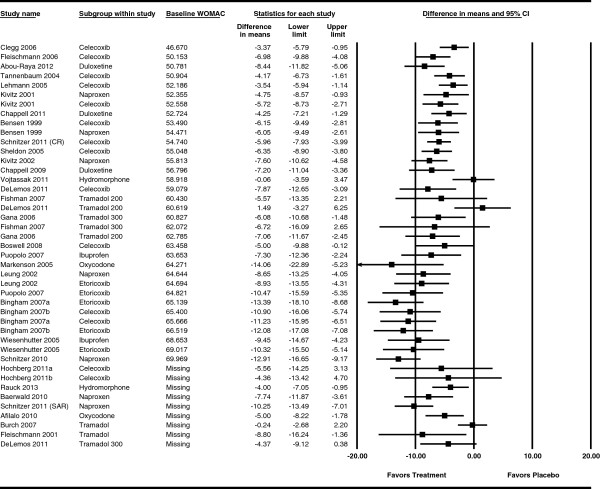
**Forest plot by baseline WOMAC showing difference in change from baseline.** Note: the lower limit in the Markenson study extends beyond the -20.00 scale of the plot.

**Figure 5:**
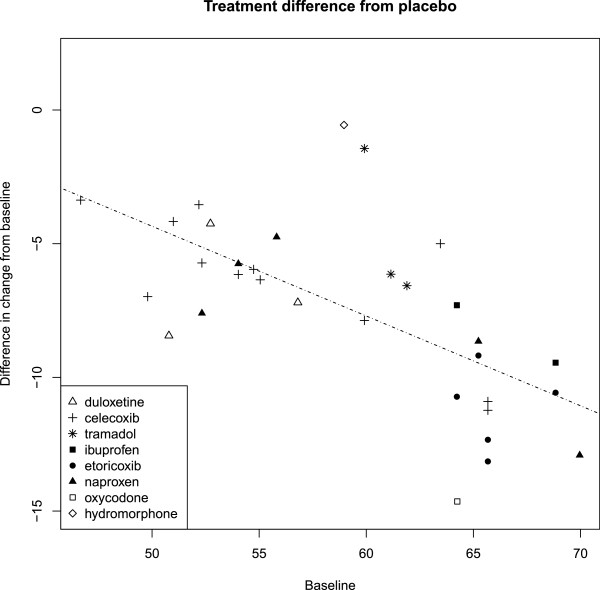
Correlation between baseline WOMAC score and the relative effect of active treatments and placebo.

To explain heterogeneity/inconsistency, we graphically explored the association of relative effect of the active treatment versus placebo with study-level covariates. Forest plots were generated for each population and study characteristic showing the difference between placebo and treatment of the change from baseline, ordered by the value of the characteristic (see Additional files 1, 2, 3, 4, 5, 6, 7, 8, 9, 10, 11). Figure 4 is the forest plot for baseline WOMAC scores. A visual association was indicated between baseline and change from baseline scores, with a higher baseline score associated with a larger negative (improved) change from baseline. Figure 5 is a verifying scatter plot showing the trial-level baseline WOMAC scores between 45 and 70 and the relative treatment effect appearing to increase as the trial-level baseline increases. A frequentist meta-regression confirmed an association between the baseline and change from baseline scores (p < 0.0001) with an R^2^ of 0. 573, indicating much of the observed improvement in symptoms was associated with a higher baseline level of symptoms.

Bayesian meta-regression models including study-level covariates were used to evaluate the extent to which covariates accounted for heterogeneity of treatment effects. Three models including study-level covariates yielded lower, similar DICs. (See Table 5). The model including the baseline score yielded both the lowest DIC and a substantially smaller SD of heterogeneity. Therefore, the model including the baseline score was preferred. Adjusted for baseline score, credible intervals of all treatments but tramadol and hydromorphone included zero, indicating no evidence of difference from duloxetine. In the cases of tramadol and hydromorphone, duloxetine demonstrated evidence of a clear advantage. When adjusted for baseline, the probability of duloxetine being superior increased for naproxen (19% to 57%), ibuprofen (28% to 82%), and etoricoxib (4% to 38%), but went down for oxycodone (41% to 15%).

**Table 5:** **Comparison of Bayesian models**^**a**^

	**Random effects**	
**Model**	**DIC**	**Heterogeneity SD**
Without adjustment	**128.29**	1.62
Without adjustment excluding studies with no baseline score	**107.00**	1.53
With adjustment		
Baseline	**93.85**	0.59
Flare	**105.32**	1.52
Analgesic use	**105.88**	1.09

^a^A lower DIC indicates a better fit of the model. A difference of 3 in the DIC between 2 models is usually meaningful [80].

## Discussion

Our analysis employed the WOMAC, a common instrument in OA trials, with subscales for function, pain, and stiffness. It is, therefore, a broader measure of OA health than instruments that focus solely on pain. Randomized controlled trials and meta-analyses in OA commonly focus on the difference between the treatment and placebo arms of improvement from baseline to endpoint. Although a commonly reported measure in meta-analysis is the standardized mean difference Cohens *d*, we chose to report the unstandardized total WOMAC score, as it is a more meaningful outcome to clinicians. In the absence of consistent statistical significance, clinical relevance was not discussed. Because OA is a chronic condition, studies were included only with a treatment duration of at least 12 weeks, the current recommended minimum duration of confirmatory chronic pain trials [30]. This has not been universal practice in other meta-analyses of OA [8-11,15-17].

With our choice of the WOMAC composite score as the outcome of interest, we chose a continuous endpoint (mean and standard deviation) rather than a dichotomous variable. It is recognized that others recommend the use of dichotomous variables (eg, 50% reduction in pain score) for evaluation of chronic pain trials. This recommendation is based on the benefits of treatment being frequently unequally distributed, typically presenting as a u-shaped distribution [81]. The WOMAC, however, is rarely reported in this manner, and our aim was to report the broader definition of health that the WOMAC encompasses, rather than pain alone.

Song et al. [41] suggests that judicious use of meta-analytical methodology can come to similar results as direct head-to-head evidence. It is frequently not possible, however, to fully account for differences in patient populations, the impact of different trial designs, and additional hidden confounders. For example, some of the trials applied flexible dose regimens (including 1 duloxetine trial) while others applied fixed dose regimens; this could impact comparative results. Enriched enrollment, a treatment run-in after screening to titrate patients up to optimal tolerability, is frequently used in opioid trials due to their well-known dosing requirements. NSAID trials, on the other hand, tend to exclude patients with a known bleeding risk or cardiovascular risk factors due to NSAIDs’ known safety profile. In the case of duloxetine, and in contrast to most other trials, a washout of previous NSAIDs was not enforced. Patients in duloxetine trials were allowed to continue (but not increase) treatment with NSAIDs with a higher proportion of patients receiving NSAIDs in placebo arms. Because this design feature only applied to duloxetine trials, they could not be accounted for overall. Such aspects can limit the interpretation and generalizability of meta-analytic results.

Statistical analyses were performed using both frequentist and Bayesian methods. Frequentist methods have the advantage of using more familiar concepts and terminology. Bayesian network meta-analysis methods have the advantage of using all the data available, such as arms from active treatment controlled trials. In this study both methods produced similar results.

Our results mirror similar findings from previous studies. A 1997 study could not recommend a choice of NSAID therapy [21]. A more recent meta-analysis commissioned by NICE did not find a statistically significant difference among NSAIDs [82]; guidelines treat NSAIDs as a class differentiated primarily by adverse events [2,3]. A meta-analysis of the short-term efficacy of treatments for OA of the knee found no statistical difference in pain relief between NSAIDs and opioids [6]. For duloxetine, our analysis repeats findings from previous studies in other pain indications. For both DPNP and fibromyalgia, duloxetine has been shown to be of similar efficacy to alternative treatment options [83,84]. Our study found a significant relationship between baseline symptoms and the magnitude of treatment effect. The related issue of the influence of flare design in trials of NSAIDs has previously been noted [7,85].

A limitation of this meta-analysis was the low number of studies available for analysis. Four or more studies were available for celecoxib, naproxen, tramadol, and etoricoxib. For all other treatments, 3 or fewer studies were found. Eight studies were omitted from the Bayesian adjusted for baseline WOMAC analysis, due to the omission of baseline scores in study publications. These numbers were, however, similar to several other meta-analyses in OA [7,8,18,21]. Limiting the literature search to English language publications may have lead to missed RCTs. However, a study examining the effect of an English-language restriction in systematic reviews and meta-analyses found no evidence of bias as a result of the restriction [86]. The funnel plot suggests that publication bias, if any, was towards the exclusion of statistically non-significant studies, further supporting our findings of no difference among comparators. Another limitation of this study is the potential for ecological fallacy associated with patient level characteristics. For example, the mean baseline WOMAC score used in the regression analysis could represent a wide variety of patient level baseline scores. A study by Lange et al. [13] points out that imputed data may bias results, showing benefit of treatment where no benefit is seen in the non-imputed data. Thus, the imputation methods used in several of the included studies could have introduced bias in the results However, its reported effect size seems to be in the range of alternative opioid treatment options such as tramadol or oxycodone [50,87].

## Conclusions

This meta-analysis found no difference between duloxetine and other post-first line oral treatments for OA in the total WOMAC score after approximately 12 weeks of treatment in a consistent manner. Etoricoxib was more effective than duloxetine in the frequentist analysis and resulted in a 96% probability of being better than duloxetine in the nonadjusted Bayesian analysis. After adjustment for baseline pain score, however, duloxetine showed evidence of superiority to both tramadol and hydromorphone, but not for the other treatments, including etoricoxib.

## Abbreviations

DIC: Deviance information criteria; DPNP: Diabetic peripheral neuropathic pain; NICE: National Institute for Health and Clinical Excellence; NSAID: Non-steroidal anti-inflammatory drug; OA: Osteoarthritis; OARSI: Osteoarthritis Research Society International; RCT: Randomized controlled trials; SD: Standard deviation; SNRI: Serotonin and norepinephrine reuptake inhibitor; VAS: Visual analogue scale; WOMAC: Western Ontario and McMaster Universities Index.

## Competing interests

J Myers, R Wielage, and J Gahn are employees of Medical Decision Modeling and were contracted by Eli Lilly and Co. to conduct this study. B Han, K Price, M Paget, and M Happich are employees of Eli Lilly and Co or its subsidiaries.

## Authors’ contributions

All authors significantly contributed to the concept and design of the study or data acquisition or analysis or interpretation, and drafting and revising the article. All authors read and approved the final manuscript.

## Pre-publication history

The pre-publication history for this paper can be accessed here:

http://www.biomedcentral.com/1471-2474/15/76/prepub

## Supplementary Material

Additional file 1 **Forest plot by washout showing difference in change from baseline.** Note: the lower limit in the Markenson study extends beyond the -20.00 Scale of the plot. Click here for file

Additional file 2 **Forest plot by concomitant analgesics showing difference in change from baseline. **Note: the lower limit in the Markenson study extends beyond the -20.00 Scale of the plot. Click here for file

Additional file 3 **Forest plot by flare requirement showing difference in change from baseline.** Note: the lower limit in the Markenson study extends beyond the -20.00 Scale of the plot. Click here for file

Additional file 4 **Forest plot by mean age showing difference in change from baseline.** Note: the lower limit in the Markenson study extends beyond the -20.00 Scale of the plot. Click here for file

Additional file 5 **Forest plot by duration of OA showing difference in change from baseline.** Note: the lower limit in the Markenson study extends beyond the -20.00 Scale of the plot. Click here for file

Additional file 6 **Forest plot by site of OA showing difference in change from baseline.** Note: the lower limit in the Markenson study extends beyond the -20.00 Scale of the plot. Click here for file

Additional file 7 **Forest plot by percentage women showing difference in change from baseline.** Note: the lower limit in the Markenson study extends beyond the -20.00 Scale of the plot. Click here for file

Additional file 8 **Forest plot by enriched enrollment showing difference in change from baseline.** Note: the lower limit in the Markenson study extends beyond the -20.00 Scale of the plot. Click here for file

Additional file 9 **Forest plot by chonic pain definition showing difference in change from baseline.** Note: the lower limit in the Markenson study extends beyond the -20.00 Scale of the plot. Click here for file

Additional file 10 **Forest plot by missing imputation technique showing difference in change from baseline.** Note: the lower limit in the Markenson study extends beyond the -20.00 Scale of the plot. Click here for file

Additional file 11 **Forest plot by quality assessment score showing difference in change from baseline.** Note: the lower limit in the Markenson study extends beyond the -20.00 Scale of the plot. Click here for file
