# Supplementary figures and images for: The efficacy of duloxetine, non-steroidal anti-inflammatory drugs, and opioids in osteoarthritis: a systematic literature review and meta-analysis
Source: BMC Musculoskelet Disord. 2014 Mar 11;15:76. doi: 10.1186/1471-2474-15-76 (PMC4007556; doi:10.1186/1471-2474-15-76)

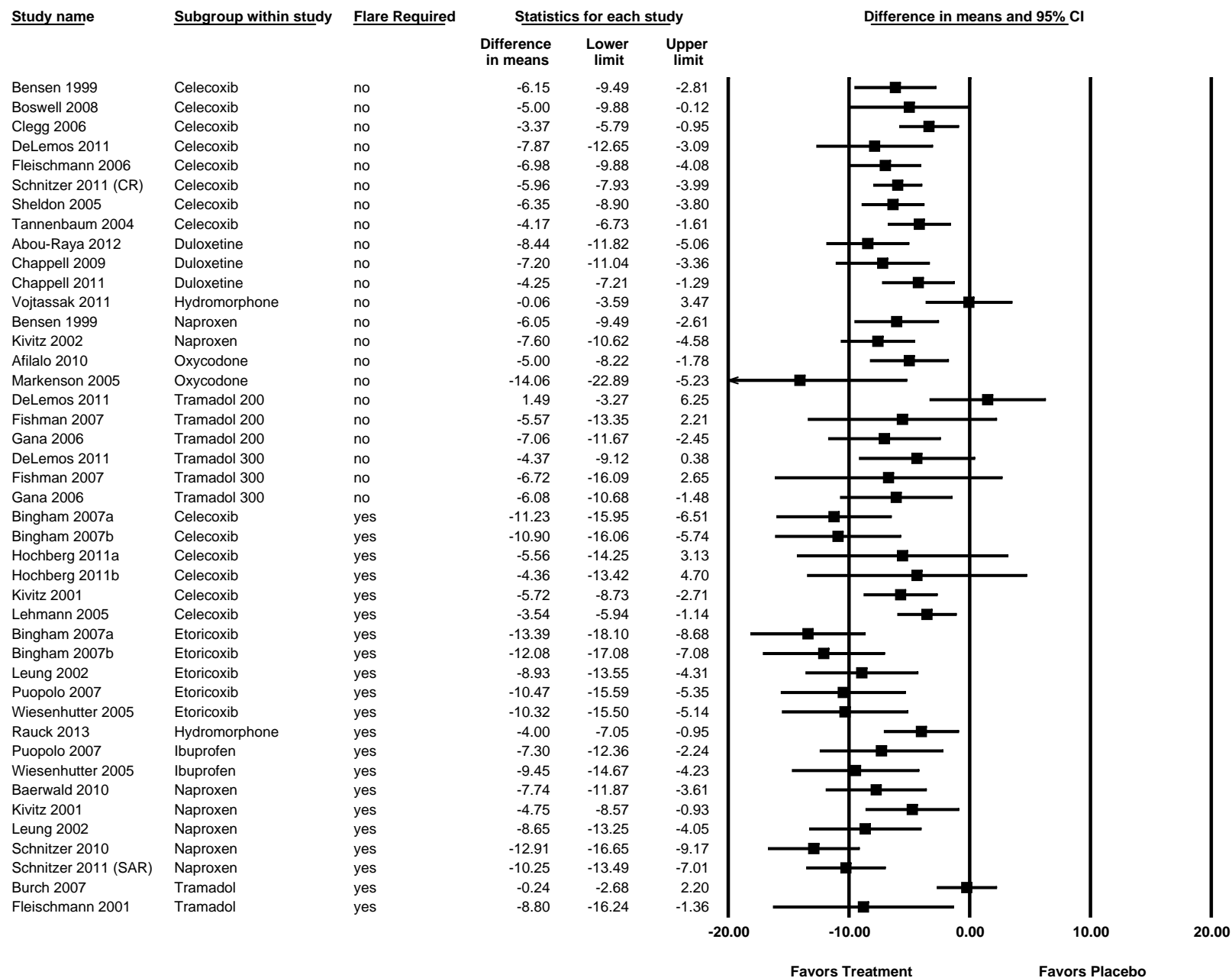

Supplement: Additional file 1 — Forest plot by washout showing difference in change from baseline. Note: the lower limit in the Markenson study extends beyond the -20.00 Scale of the plot. [file 1471-2474-15-76-S1.pdf]

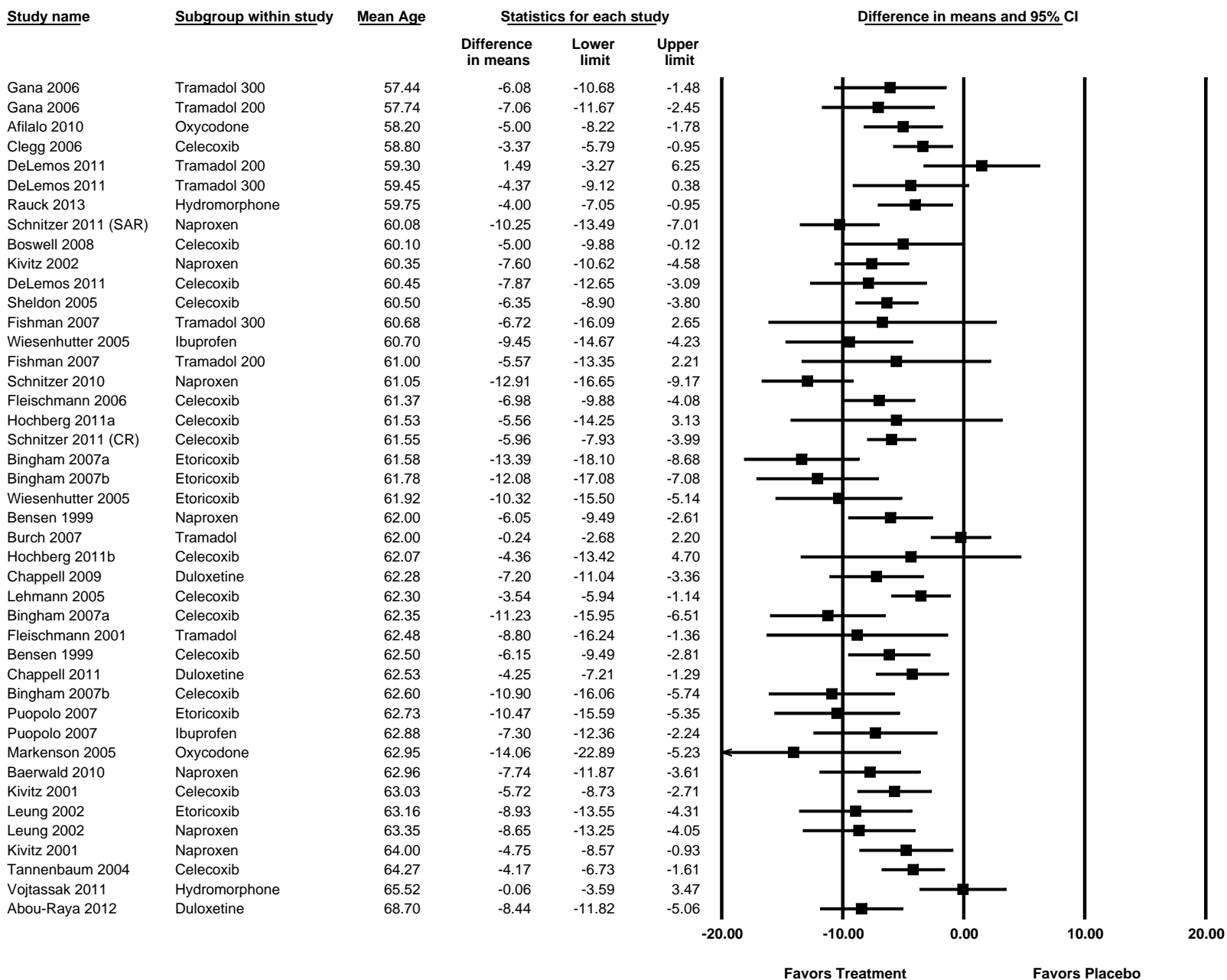

Supplement: Additional file 2 — Forest plot by concomitant analgesics showing difference in change from baseline. Note: the lower limit in the Markenson study extends beyond the -20.00 Scale of the plot. [file 1471-2474-15-76-S2.pdf]

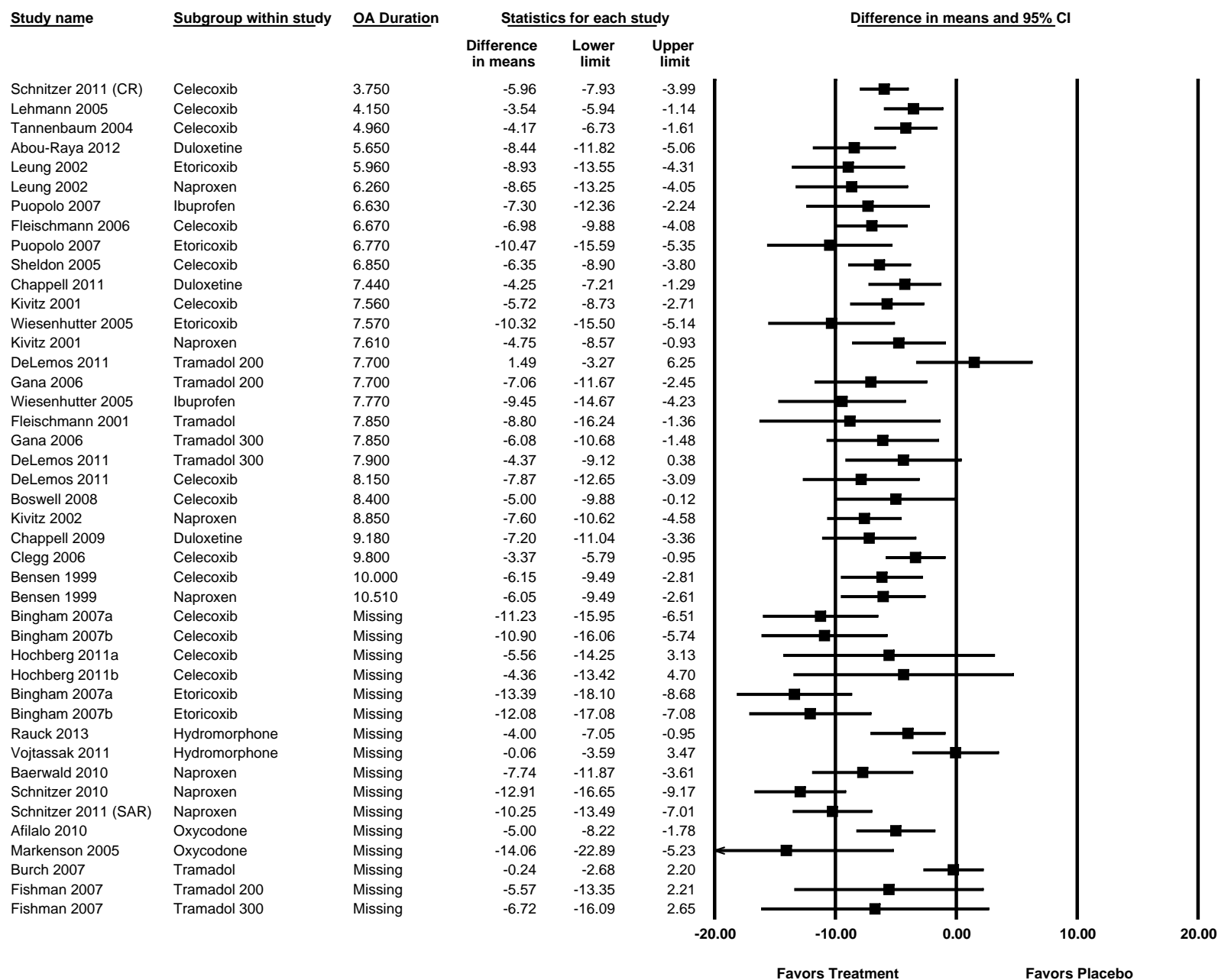

Supplement: Additional file 3 — Forest plot by flare requirement showing difference in change from baseline. Note: the lower limit in the Markenson study extends beyond the -20.00 Scale of the plot. [file 1471-2474-15-76-S3.pdf]

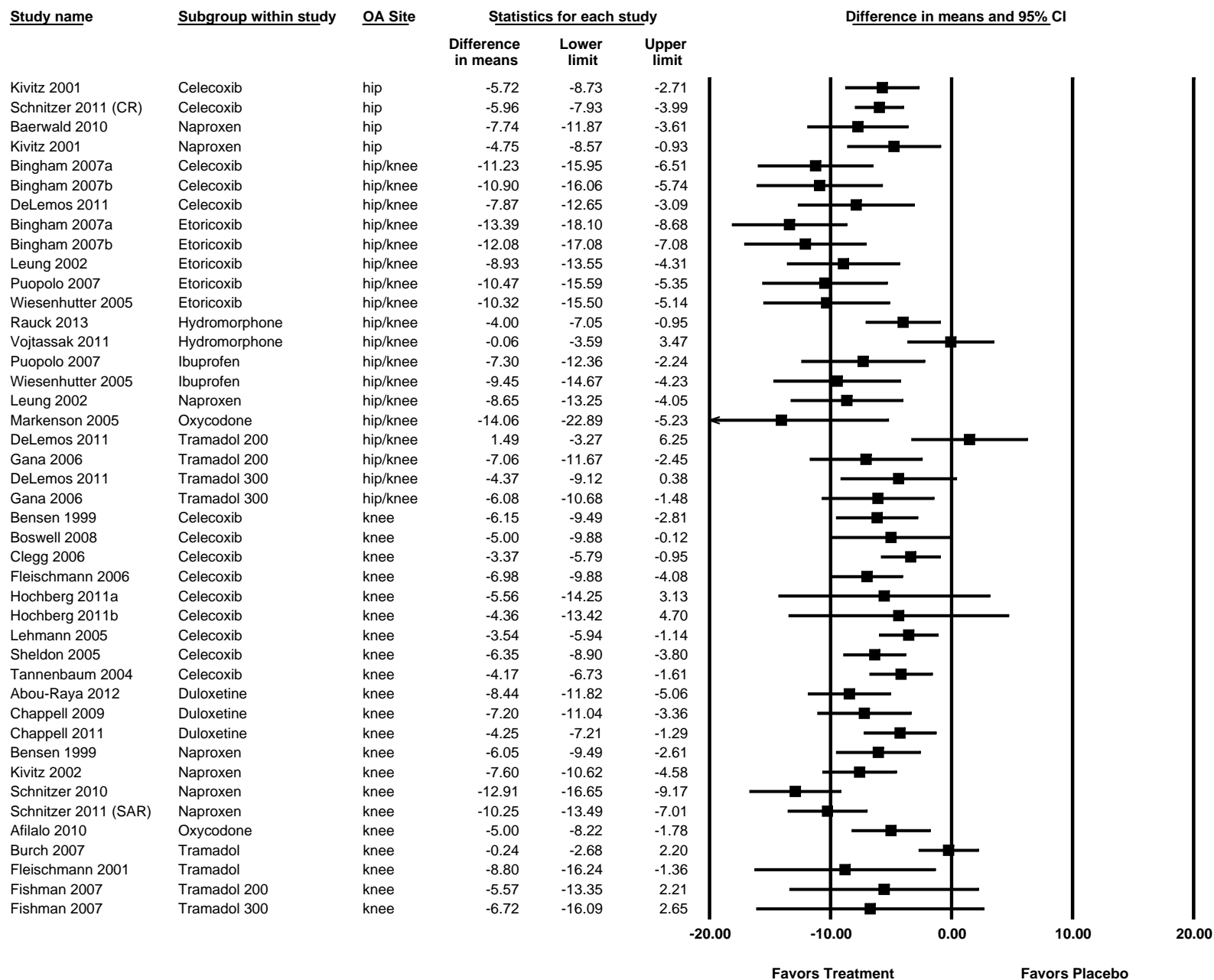

Supplement: Additional file 4 — Forest plot by mean age showing difference in change from baseline. Note: the lower limit in the Markenson study extends beyond the -20.00 Scale of the plot. [file 1471-2474-15-76-S4.pdf]

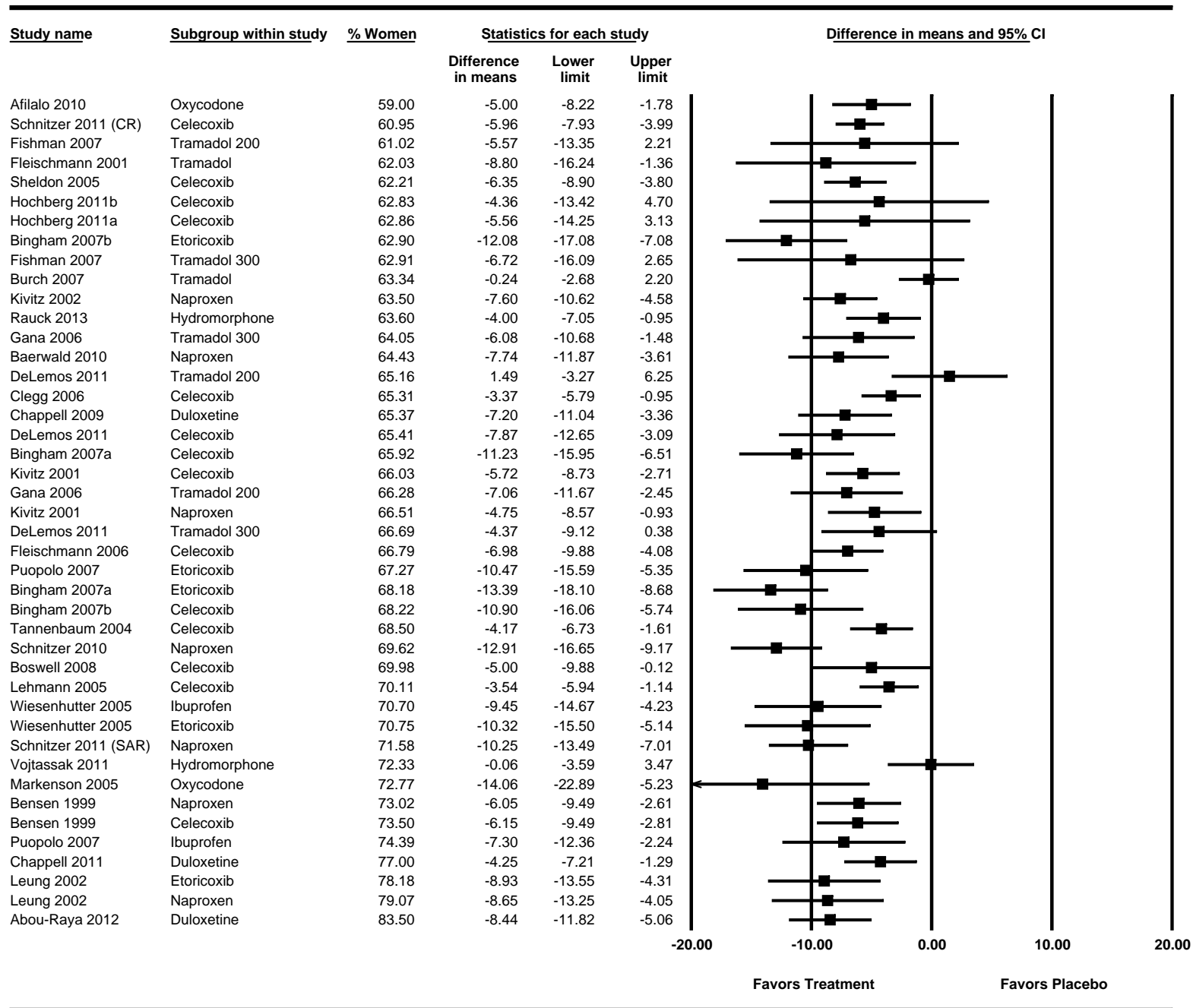

Supplement: Additional file 5 — Forest plot by duration of OA showing difference in change from baseline. Note: the lower limit in the Markenson study extends beyond the -20.00 Scale of the plot. [file 1471-2474-15-76-S5.pdf]

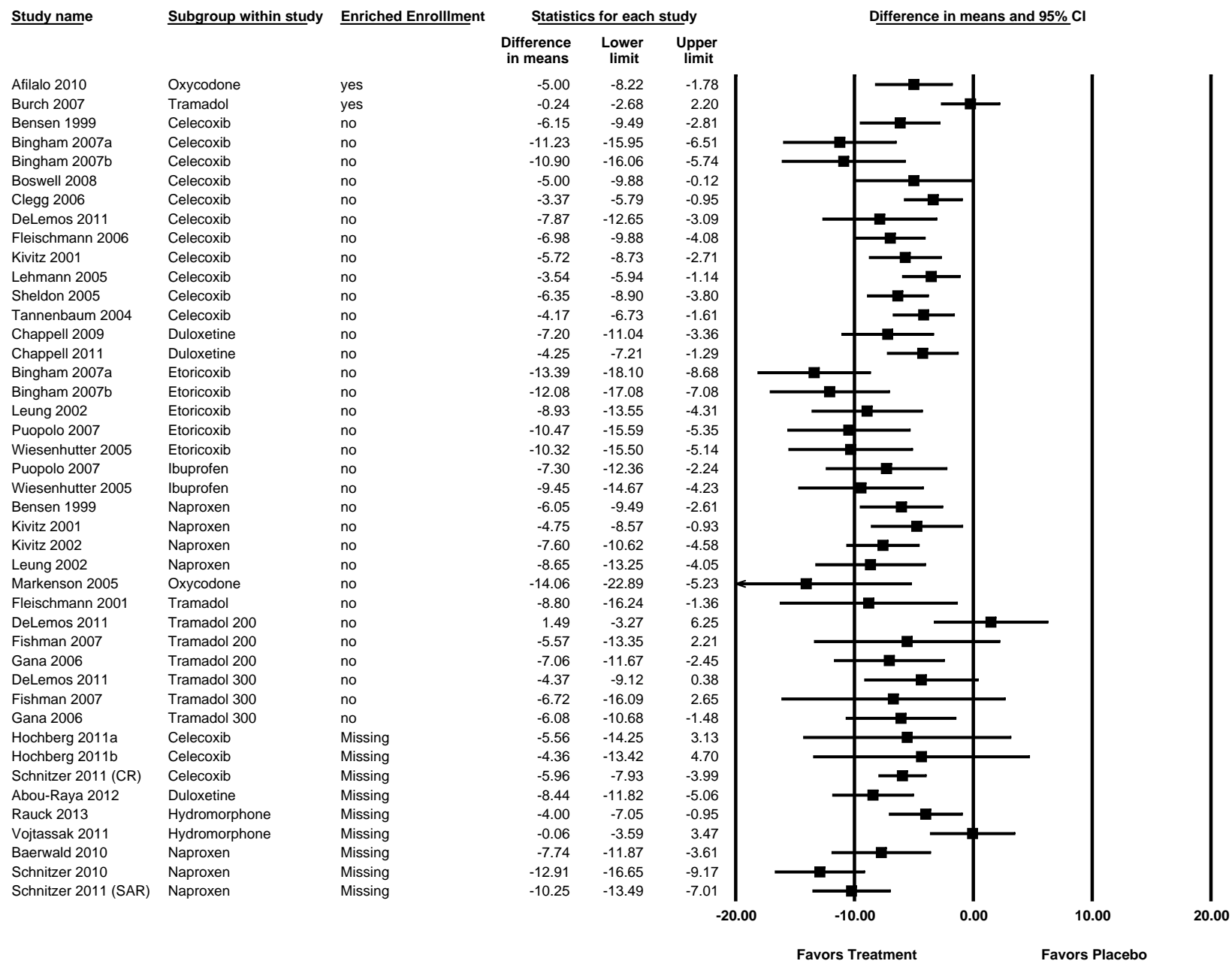

Supplement: Additional file 6 — Forest plot by site of OA showing difference in change from baseline. Note: the lower limit in the Markenson study extends beyond the -20.00 Scale of the plot. [file 1471-2474-15-76-S6.pdf]

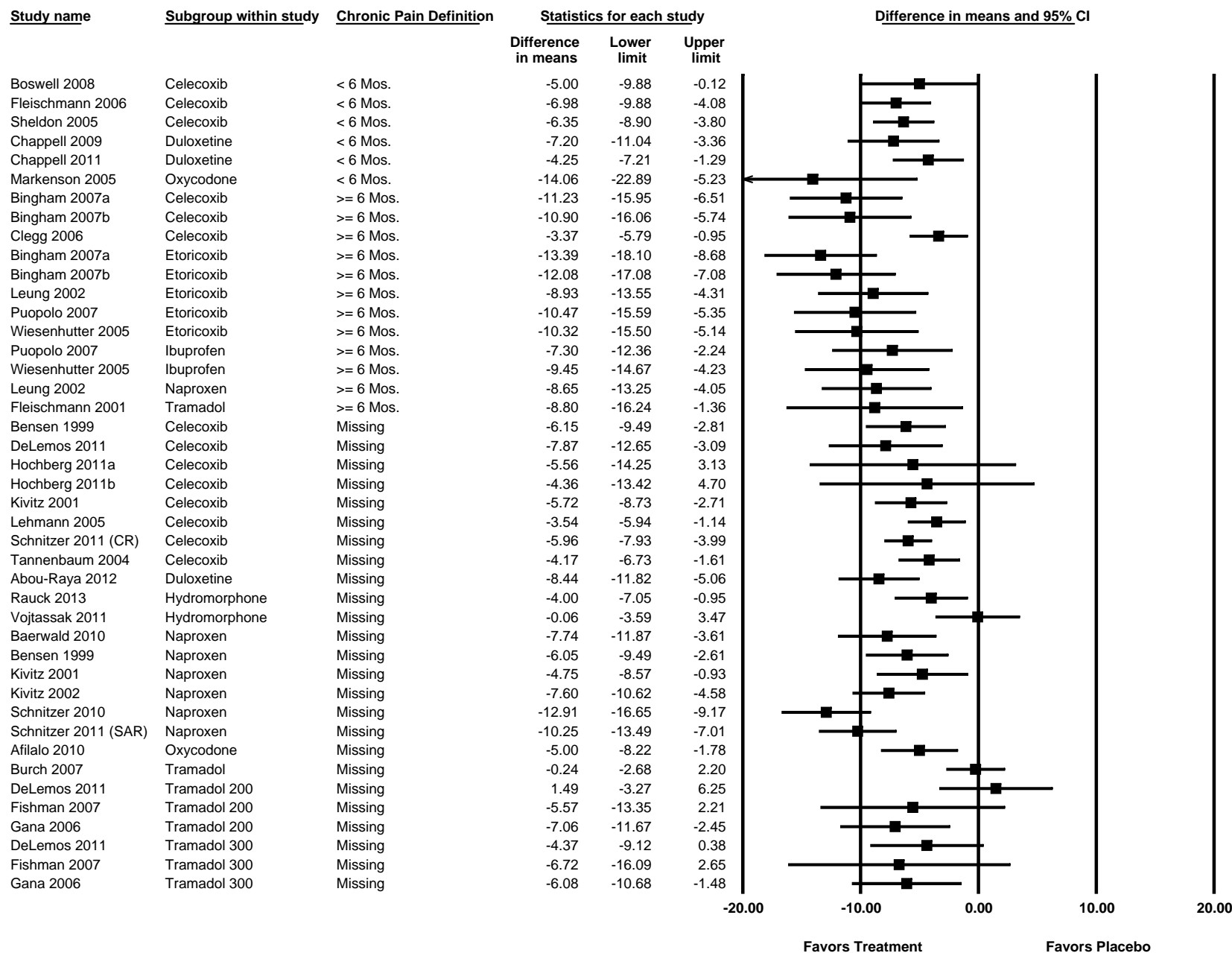

Supplement: Additional file 7 — Forest plot by percentage women showing difference in change from baseline. Note: the lower limit in the Markenson study extends beyond the -20.00 Scale of the plot. [file 1471-2474-15-76-S7.pdf]

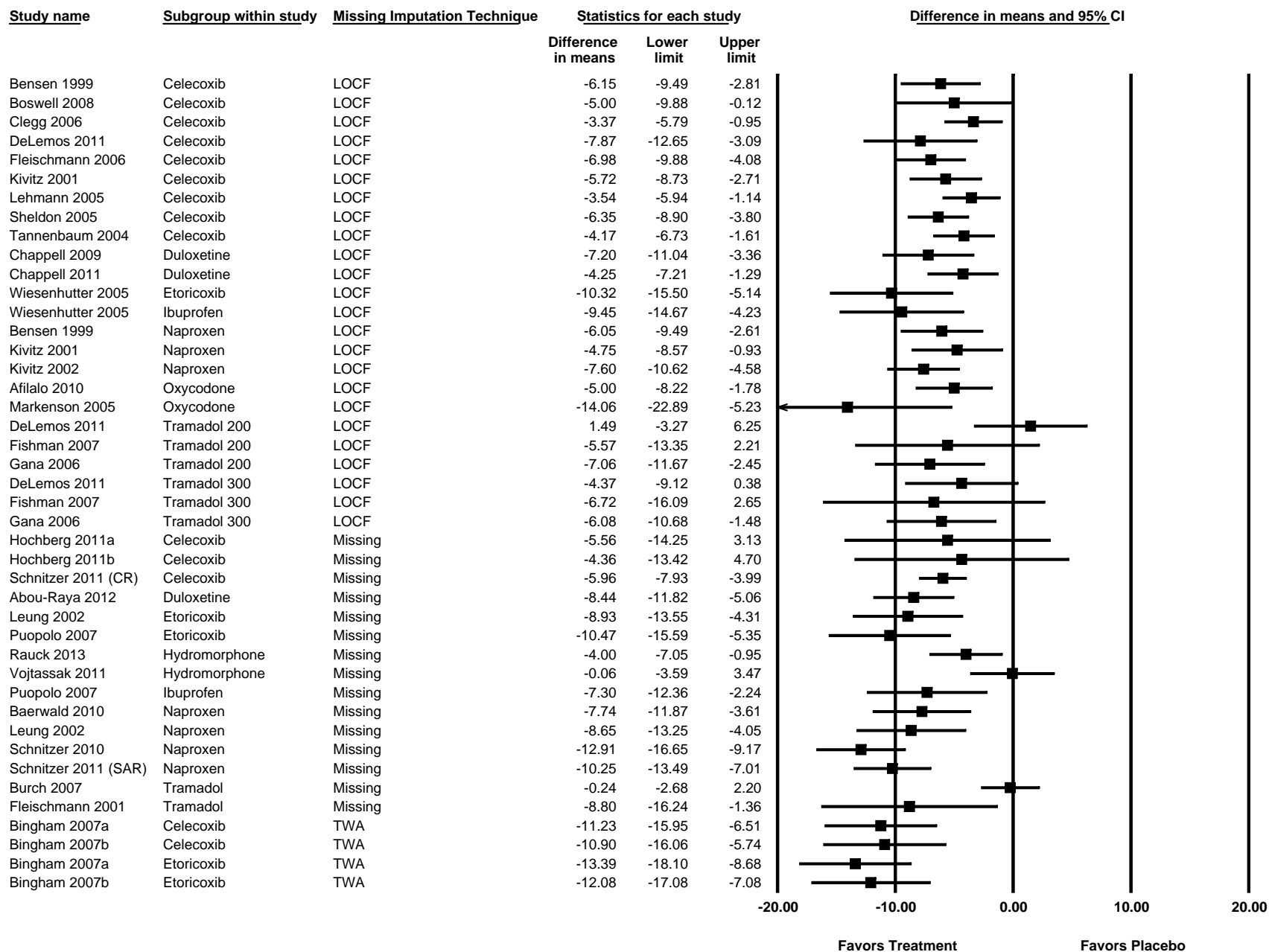

Supplement: Additional file 8 — Forest plot by enriched enrollment showing difference in change from baseline. Note: the lower limit in the Markenson study extends beyond the -20.00 Scale of the plot. [file 1471-2474-15-76-S8.pdf]

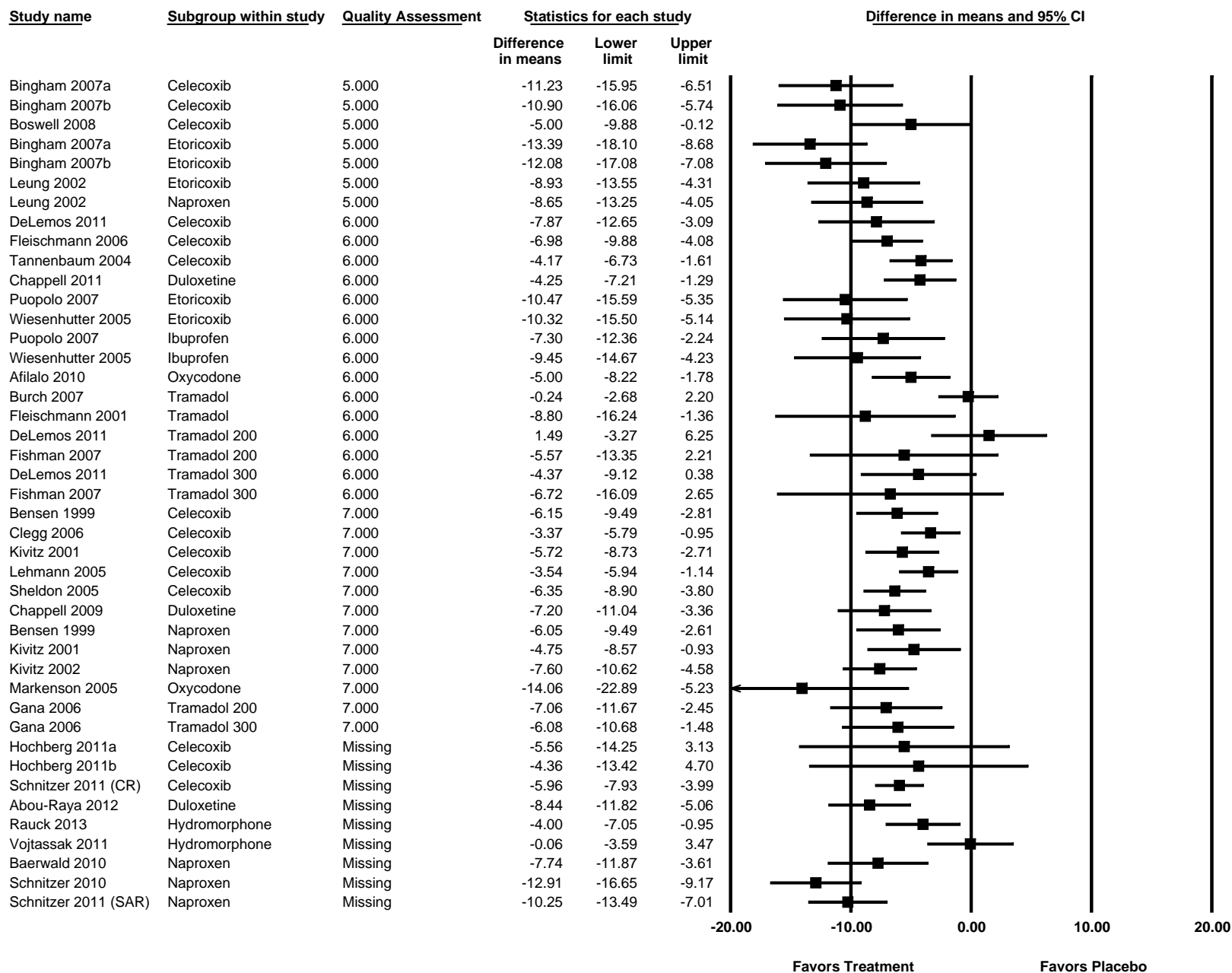

Supplement: Additional file 9 — Forest plot by chonic pain definition showing difference in change from baseline. Note: the lower limit in the Markenson study extends beyond the -20.00 Scale of the plot. [file 1471-2474-15-76-S9.pdf]
